# Aspects Towards the Anastomotic Healing in Crohn’s Disease: Clinical Approach and Current Gaps in Research

**DOI:** 10.3389/fsurg.2022.882625

**Published:** 2022-06-24

**Authors:** F.H.M. Chaim, L.M.V. Negreiros, K.M. Steigleder, N.S.N. Siqueira, L.M. Genaro, P.S.P. Oliveira, C.A.R. Martinez, M.L.S. Ayrizono, J.J. Fagundes, R.F. Leal

**Affiliations:** Inflammatory Bowel Disease Research Laboratory, Gastrocenter, Colorectal Surgery Unit, School of Medical Sciences, University of Campinas (Unicamp), São Paulo, Brazil

**Keywords:** anastomotic healing, inflammatory bowel disease, surgical complications, Crohn’s disease, anastomosis, mesenteric adipose tissue, postoperative complications/prevention & control, suture techniques

## Abstract

Anastomotic leakage is a major complication in gastrointestinal and colorectal surgery and its occurrence increases morbidity and mortality. Its incidence is even higher in Crohn’s disease surgeries. Several authors have identified factors involved in the pathophysiology of anastomotic leak in the literature, aiming to reduce its occurrence and, therefore, improve its surgical treatment. Surgical technique is the most discussed topic in studies on guiding the performance of side-to-side stapled anastomosis. Preoperative nutritional therapy also has been shown to reduce the risk of anastomotic leakage. Other factors remain controversial – immunomodulator use and biologic therapy, antibiotics, and gut microbiota – with studies showing a reduction in the risk of complication while other studies show no correlation. Although mesenteric adipose tissue has been related to disease recurrence, there is no evidence in the literature that it is related to a higher risk of anastomotic leakage. Further exploration on this topic is necessary, including prospective research, to support the development of techniques to prevent anastomotic leakage, in this way benefiting the inflammatory bowel disease patients who have to undergo a surgical procedure.

## Introduction

The inflammatory bowel disease (IBD) therapeutic arsenal has broadened in the last decades due to pharmacological advances and the development of new drugs (1). Nevertheless a considerable percentage of patients need to undergo surgical treatment one or more times during their lifetime. From 50 to 90% of Crohn’s disease (CD) patients, will require some type of surgical procedure along with medical follow-up and treatment period (2–4). The need to perform a surgical procedure is about 4 times lower (approximately 18%) among patients diagnosed with ulcerative colitis (UC) (2). And yet, the performance of surgical propaedeutic requires the implementation of procedures with greater invasiveness and risk to the patients.

Implementation of surgical treatment incurs the risk of occurrence of postoperative inherent complications, including hemorrhage, intraperitoneal collection, wound infection and dehiscence, fistulas, pulmonary complications, and thromboembolic events. Among those complications, anastomotic leakage (AL) is a major complication in gastrointestinal and colorectal surgery and its occurrence contributes significantly to the increase in morbidity and mortality (5).

The pathophysiology of CD involves the occurrence of acute complications (e.g., bleeding, bowel obstruction, perforations; severe acute colitis) and chronic complications (e.g., strictures and stenosis, internal or external fistulas, adhesions, abdominal masses) as well as disease forms refractory to pharmacological therapy. Intestinal or colon resection is the required basis of surgery in CD and the consequent need to perform an anastomosis is a common fact during surgery. Anastomosis and reoperation are intrinsically related. The realization of an anastomosis increases the risk for urgent reintervention due to AL and in long term, postoperative recurrence typically occurs at the anastomotic site (3).

The surgically related incidence of anastomotic dehiscence in the literature varies widely. In a recent observational study involving more than 36.000 subjects who submitted to surgery due to colorectal carcinoma, AL incidence was 4.1% (6). Its incidence is even higher in CD surgeries. Recent studies enrolling CD patients showed AL occurrence in 6.4% up to 14% of patients submitted to surgical treatment (7–9). Therefore, surgeons are in constant pursuit of practices to prevent AL, in this way benefiting IBD patients who have to undergo a surgical procedure.

Mesenteric adipose tissue (MAT) and its mediators are increasingly more implicated in CD pathogenesis. Recent accumulating evidence also highlights the role of creeping fat in contributing to disease recurrence, to the point that it has become a well-known feature of CD (10, 11).

There is ample work in the literature concerning the healing process of anastomosis and AL after colorectal surgery in the context of neoplastic disease. However, the information on IBD and specifically in CD surgical treatment is sparse. This article aims to discuss and summarize the main topics present in the literature and identify potential areas for future research on the subject.

Despite recent advances in gastrointestinal/colorectal surgical technique and perioperative care, anastomotic healing is still a matter of concern in CD patients submitted to surgical treatment and AL remains a major complication. Its etiology is not yet completely understood. However, it is multifactorial and not only influenced by surgery-related factors but also by factors related to the disease and its behavior.

## Surgical Technique

The decision concerning anastomotic configuration depends on the surgical team’s preference, the surgeon’s experience, the availability of surgical materials (for example staplers and surgical threads), and the financial reality of each hospital.

Historically, hand-sewn anastomosis was the most common, and these produced variations related to the surgical thread and the type of stitches used. With the development and wide use of various types of staplers, even more, possible variations were added to this debate.

**Figure 1:**
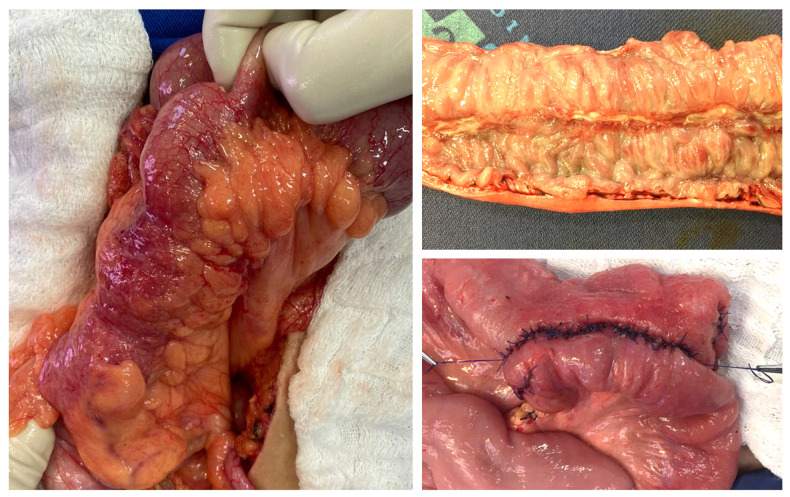
Aspects of an anastomosis followed by an enterectomy for Crohn’s disease (CD). (**A**) Surgical aspect of the ileum affected by CD. (**B**) Surgical specimen showing a longitudinal deep ulcer in the inflamed intestinal mucosa by the disease. (**C**) Side-to-side anastomosis. Source of the photographs: Colorectal Surgery Unit, Unicamp.

Side-to-side stapled anastomosis significantly reduces the incidence of short-term complications in surgical patients with CD when compared to end-to-end anastomosis (OR 0.54; 95% CI, 0.34 to 0.83). Specifically for the AL rate, side-to-side stapled anastomosis determines a decrease from 14.1 to 2% (95 percent confidence interval 1.7–22.2; *p* = 0.02). A reduction in mean postoperative hospital stays from 12.3 to 9.7 days (*p* = 0.03) was also observed (9, 12) (Figure 1A).

The technique chosen must consider the location of anastomotic performance (accessibility), the caliber of the intestinal segments (presence of edema may influence), surgical contamination, and the progress of the disease. According to the ECCO Consensus, the anastomotic diameter is an important discrimination factor that must be considered (13). Some authors suggest that for resections performed in the ileum, side-to-side anastomoses are indicated, in order to have a wider anastomotic lumen, while end-to-end anastomoses are performed in colonic segments, which have a larger caliber (14).

Due to the high recurrence rate characteristics of CD pathophysiology, repeat surgery is often required. In this sense, performing surgical procedures in CD patients with previous intestinal resection (reoperation) is an independent risk factor for AL and the number of previous resections is correlated with increased risk (8) (Figure 1B).

Recently, the laparoscopic approach has become more frequent and has become the standard of care in most situations. Robotic-assisted surgery also has been gaining acceptance in colorectal benignant and malignant surgery with intracorporeal anastomosis. Evidence on digital and robotic platform surgery applied to CD is scarce and recent, so it is still liable to selection bias. However, it seems to point to a lower occurrence of AL in patients undergoing robotic surgery (15). It has also already been verified that CD patients seem to be the most technically difficult group to apply the robotics procedures (16, 17). Despite the numerous benefits of these less invasive approaches, complications associated with stapled tissue continue to be a concern.

## Timing of Surgery

Considering that most CD patients will require surgical treatment over time (2–4), it becomes a persistent and recurrent dilemma in the daily practice of the surgeon: the decision between indicating an early surgery (incurring all the risks related to the surgical intervention) or continuing to try clinical treatments (at the risk of having to approach the patient later with an even more deteriorated clinical condition).

The literature is controversial regarding the optimal timing of surgery. Even the period that is taken into account to define a surgical intervention as early is inconstant, varying from 6 to 18 months after CD diagnosis (18, 19). Earlier surgical approaches would be related to the performance of technically easier procedures, smaller resections and consequently lower postoperative complications.

In a study performed by An et al., 31.3% of patients who were initially treated with drugs for ileocolonic CD required surgery within 5 years. In addition, patients in this cohort who underwent early surgery demonstrated a more benign course of the disease with fewer future surgical interventions and fewer hospitalizations (18). A prospective randomized controlled trial enrolling 134 localized ileocecal CD patients, demonstrated that both early and delayed resections are comparable in terms of their influence on the quality of life and that early resection is more cost-effective and associated with lower clinical recurrence (20).

Reliable predictors for the need for surgical interventions are yet to be established, to assist the surgical decision-making and individualize the treatment for each patient. Despite all the data that elucidate the advantages of performing early surgical interventions, the well-being of the patient should also be considered, especially in terms of the psychological aspects, such as anxiety, and also the consequences of surgeries such as the performance of stomas, the peri and postoperative risks, in addition to the possibility of CD recurrence even after surgical resection (21). Post-surgical complications such as infections, bleeding, anastomotic leakage, and mortality are questions that must be considered before choosing the intervention, in addition to factors such as the technical skills of the surgeons who will perform it. The final decision on the ideal moment of surgical therapy must be individualized for each patient, considering the characteristics of the disease, such as its phenotype, the risk factors involved in the process, and the patient’s opinion regarding the procedure (22).

## Medications

The indication of surgical treatment for CD patients may occur in one of two different settings: emergent operations due to acute decompensating or life-threatening events in patients without a previous diagnosis, or elective operations which are indicated due to failure of clinical treatment. This distinction is relevant because in the latter situation, patients are in use of one or more drugs and it may influence the anastomotic healing process.

Conventional treatment has evolved to induce and maintain remission, thus avoiding complications, such as the need for surgical interventions. If this objective is not achieved, and the patient has to undergo surgical procedures, an evaluation of preoperative, perioperative, and postoperative medication uses is needed, and their implications for an increased risk of postoperative complications must be considered (23, 24).

Corticosteroids are anti-inflammatory drugs and have been widely used in the treatment of CD since 1950. Their use is only indicated for induction and not for maintenance of remission (25–28). However, they can have a negative influence, generating surgical complications and ineffective healing (29, 30). For elective surgeries, there is still no consensus regarding the recommendation to reduce the doses of corticosteroids before surgery, and in the studies that recommend doses reduction the preoperative interval varies from 3 to 6 weeks (13, 23, 31, 32).

Immunomodulators have been widely used for maintenance of remission or in conjunction with biological therapy to decrease surgical needs in CD patients. To date, studies have shown that its use does not adversely affect postoperative results (33, 34). Therefore, it is recommended to discontinue thiopurines on the day of surgery and reintroduce them along with all oral medications, if renal function remains normal. Methotrexate can be maintained pre-and post-operatively when the patient does not have an infection or renal failure (23, 35).

Biological medications, used in the treatment of various immune-mediated disorders, have revolutionized the treatment of CD. These medications are effective in containing inflammation and mucosal healing and reducing hospitalization and surgery rates (36–38). Despite the benefits established in the literature, this therapy has already been shown to be associated with an increased risk of postoperative septic complications in traditional abdominal surgeries for CD. Therefore, it is, recommended for elective surgeries to respect the longest possible interval between doses (e.g., 4 weeks for infliximab and a minimum of 2 weeks for adalimumab) (23, 39). Reintroduction is recommended approximately 3–4 weeks after definitive healing of the anastomosis (13).

**Figure 2:**
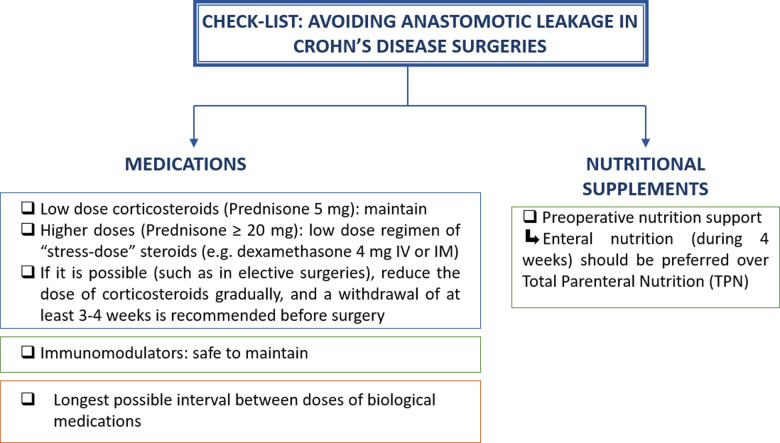
Check-list to guide surgeons to avoid complications in Crohn’s disease abdominal surgeries that require intestinal resection and anastomosis.

Figure 2 summarizes the recommended management of immunosuppressors and biological therapy in CD patients who undergo abdominal surgery.

## Nutritional Aspects

Aiming to reduce postoperative complications, surgeons have been making efforts to act beyond the surgical technique itself and control multidisciplinary perioperative issues to obtain better surgical results. In this sense, there is evidence that perioperative nutritional aspects are an important predictor of risks and complications (40).

Preoperative nutritional status is directly related to anastomotic healing since malnutrition directly interferes with collagen synthesis and fibroblast proliferation (40). Impaired preoperative nutritional status defined as anemia (hemoglobin ≤10.0 g/dL), or hypoproteinemia (albumin ≤3.2 g/dL) is significantly associated with complications (41). Preoperative hypoalbuminemia is an independent risk factor for intraabdominal septic complications after ileocolic resection (42, 43). These changes in perioperative albumin levels may reflect the severity of systemic inflammation, protein-losing enterocolopathy, malnutrition, or concurrent liver dysfunction (42).

In the postoperative period, malnutrition characterized by hypoalbuminemia can be a tool to identify patients at high risk of AL. Mean serum albumin levels on postoperative days 1 and 3 are significantly lower in patients that will present AL (44).

Even so, hypoalbuminemia is not a direct marker of preoperative nutritional status, nor is it the only biomarker that can be used for this purpose (42, 45). Several blood biomarkers, in addition to albumin, can be useful biochemical indicators to characterize malnutrition, even in the presence of chronic inflammation (e.g., pre-albumin, hemoglobin, total cholesterol, and total protein) (45). When malnutrition is detected in preoperative patients, nutritional optimization by enteral nutrition (EN) or total parenteral nutrition (TPN) is necessary to improve the surgical results reducing the overall rate of postoperative complications including AL (46). Nonetheless, further studies are needed to evaluate the best malnutrition biomarkers directly related to AL occurrence in CD surgeries.

The use of nutritional therapy (NT) has shown promise in modulating the inflammation in CD patients who required surgical resection, whether in exclusive or partial use (47, 48). Guo et al. evaluated the use of NT using exclusive EN with a polymeric formula that was infused continuously through a nasogastric tube (40). Two weeks of preoperative EN significantly increases albumin level, decreases C reactive protein, and also decreases AL incidence (48). Nutritional therapy in CD can reduce inflammation of intestinal and mesenteric fat, by reducing the expression of pro-inflammatory cytokines such as IL-1beta, IL-6, TNF-alpha, and leptin and increasing anti-inflammatory cytokines such as adiponectin (46, 49). This anti-inflammatory effect also may be able to improve wound healing ability (48).

The European Society for Parenteral and Enteral Nutrition has published guidelines addressing preoperative nutrition on IBD. Patients with severe nutritional risk (weight loss >10%–15% within six months; BMI <18.5 kg/m^2^; serum albumin <3.0 g/dL with no evidence of liver or kidney dysfunction) should have surgery delayed for 7–14 days whenever possible (50). This period needs to be used for EN supplementation, and if there are any contraindications such as intestinal obstructions or ileum or high output fistulas, TPN should be indicated (51, 52). Although it does not specifically address only AL, a recent and comprehensive meta-analysis enrolling 1111 CD patients showed that preoperative nutritional supplementation through the use of EN is a positive prognostic factor and significantly reduced the overall rate of postoperative complications from 73.2% to 21.9% (OR = 0.09, 95% CI, 0.06–0.13, *p* < 0.001), when compared to the group that received standard nutritional care. However, a consensus about differences in specific nutrition formulations and the duration of enteral nutrition is yet to be achieved (52). Although the use of TPN did not reach statistical significance, it pointed to a trend in reducing postoperative complications.

**Figure 3:**
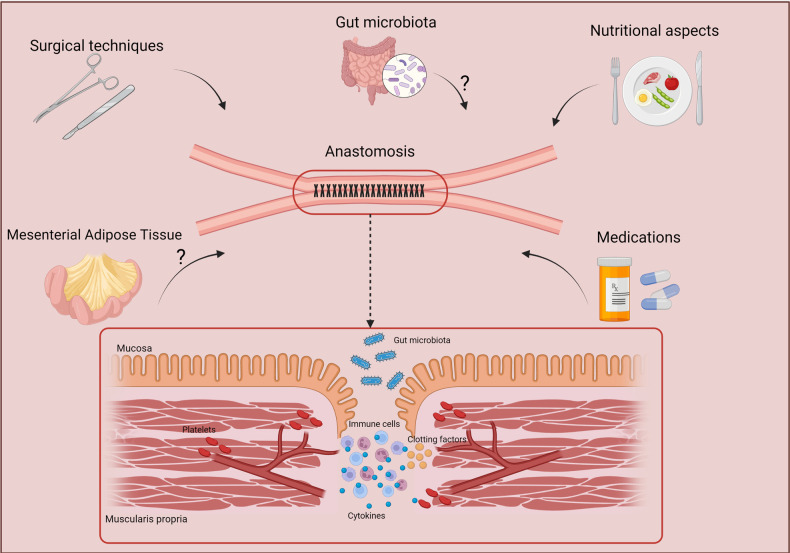
Factors that may influence the effective healing of anastomosis. Several factors may act directly on the correct healing of anastomoses, such as nutritional factors, intestinal microbiota, medications, and the tissues nearby the intestinal affected area such as the mesenteric adipose tissue. Created with BioRender.com.

Figure 2 also summarizes the recommended management of nutrition in CD patients who undergo abdominal surgery (Figure 3).

## Gut Microbiota

Gut microbiota has gained extensive importance in IBD pathophysiology and, thus, intraluminal microbes may also interact and have a significant influence on the anastomotic healing and consequently the risk of AL occurrence. An impaired permeable intestinal barrier can lead to exposure of the microbiota and its metabolites to various components, therefore becoming triggers for changes in physiology and the immune responses (53, 54).

Dysbiosis, a loss of balance of the intestinal microbiota, is associated with several diseases, including CD, presenting an increase in the number of pathogenic bacteria and reducing the proportion of the beneficial ones (55, 56). Thereby, CD patients have a reduction in bacteria of the genus *Firmicutes* (57), and an increase in specimens of the *Enterobacteriaceae* family (58) in their stool, when compared with healthy individuals.

Several studies have shown a relationship between intestinal microbiota and the healing process of anastomoses. In addition, external environmental factors such as comorbidities and surgeries directly impact the microbiota, being able to select and boost colonization by species considered more aggressive (59, 60). One of the products derived from the metabolism of intestinal bacteria is butyrate, a short-chain fatty acid, which acts as a source of energy for epithelial cells while decreasing their permeability and increasing the stiffness of junctions. In experimental studies, oral and rectal administration of butyrate during the perioperative period of intestinal resection demonstrated that the performed anastomoses were firmer (61). Similarly, it was demonstrated that the oral use of pectin in the rat model led to an increase in the production of short-chain fatty acid, thus contributing to accelerating the healing of the performed anastomoses (62). Although dietary factors are implicated in CD pathophysiology, the effects of dietary interventions based on fiber substrates with pectin supplementation are uncertain (63, 64).

Studies were also performed in humans to analyze the direct influence of the microbiota on the anastomoses. Comparing the microbiota of patients who had AL with that of patients whose anastomoses healed with no sign of leakage, it has been shown that patients with AL had lower microbial diversity and abundance in the *Lachnospiraceae* and *Bacteroidaceae* families, which may be directly related to AL due to its association with mucin-degrading bacteria (65).

Despite the availability of several approaches to modulation of gut microbiota for therapeutic benefit in CD patients (e.g., removal with antibiotics, replacement and reset with specific or multiple-bacteria probiotics), results to date have not found consistent evidence for the effectiveness of probiotics in these patients for the prevention of postoperative complications. Although it has been demonstrated that probiotics improve the bacterial variety, decrease the growth of pathogenic bacteria, and enhance the intestinal barrier, a detailed study demonstrating its benefit in clinical practice still lacking (66).

## Mesenteric Adipose Tissue

In recent years, MAT has gained increasing importance in the research of CD pathophysiology. Starting from the initial observation that chronic CD patients with transmural inflammation have MAT increasing nearby the affected intestinal area, alterations were found among the numerous functions of this tissue. It at least partially justifies the variations, the severity and contributes to the understanding of the disease.

Given the numerous mesentery intraoperative features found by surgeons (e.g., signs of inflammation, mesenteric thickening, edema extending circumferentially, presence of granulomata, increased mesenteric lymphatic vessel and lymph nodes density), it was postulated that inflammatory activity would result of the convergence of inflammatory inputs coming from both the submucosa and the mesentery. As a consequence, new mesenteric excision-based surgical strategies were formulated aiming to improve postoperative outcomes (67, 68).

Results from an international, multicenter, randomized controlled trial protocol comparing mesenteric excision or conservative limited resection in small intestine CD surgery, suggest that the inclusion of the mesentery during bowel resection improves the natural history of postoperative clinical and surgical recurrence of CD (11). Similarly, in another study, the mesentery resection technique was an independent determinant of postoperative recurrence rate in ileocolic resection for CD and the adoption of mesentery resection reduced the reoperation rate from 40% to 2.9% (69). Although mesenteric excision in CD may reduce postoperative disease recurrence, there is no robust data about the occurrence of morbidities, such as AL in cases that would require a larger resection of this tissue.

The extension of mesenteric resection has been evaluated to determine the effects of a more extensive or limited excision on early postsurgical outcomes. Available data until now shows that extensive mesenteric resection is associated with a longer postoperative recurrence-free survival time (70). The involvement of MAT in CD is a fact. However, remains uncertain adequate excision extension and is still to be determined effects of MAT resection on the early postoperative period and AL rates.

A configuration of antimesenteric hand-sewn functional end-to-end anastomosis nominated “Kono-S” has been developed in 2003, based on cautious mesenteric excision, a stabilizing structure, and a wide anastomotic lumen. Since its introduction in Japan, its performance has spread around the world with cumulative evidence of favorable results. Kono-S technique is associated with a lower recurrence rate when compared to the standard hand-sewn end-to-end anastomosis (69, 71, 72). A recent meta-analysis enrolling 676 CD patients not only demonstrated a very low clinical and endoscopic recurrence rate (5% CI, 0.00–0.15) but also a small incidence of anastomotic leakage (1% CI, 0.00–0.03) (73). In addition, depending on the affected topography of the gastrointestinal tract, the surgical approach will involve the resection of specific intestinal segments and, consequently, the performance of the corresponding anastomosis. In this context, the literature data favors the Kono-S anastomosis even further when performing an ileocolic anastomosis (73, 74).

## Final Considerations

The pursuit for the ideal anastomosis (technically easy, without the need for expensive materials, with a low rate of AL and low recurrence rate) is still ongoing. Based on data available until now, it is recommended to perform side-to-side anastomosis to obtain lower rates of AL. The benefits of pharmacological therapies to CD patients are irrefutable but in the perioperative setting, they may worsen the anastomotic healing process. In this sense, it is of extreme importance that the surgical team evaluate the medications that the patient uses in the context of elective surgeries to decide on their suspension or maintenance. Moreover, preoperative nutritional therapy impacts surgical outcomes by reducing AL rates. It is yet to be established whether there is a specific biomarker endpoint to be accomplished before performing elective surgeries to get lower AL rates.

Therefore, this review aimed to contribute to a better understanding of the anastomotic healing in CD patients and to highlight the factors that directly may affect it. CD is a chronic inflammatory disease that still has no cure, and many patients need surgical interventions at least once during the course of the disease. All these factors potentially involved with anastomotic healing are important and need to be analyzed carefully to provide a better outcome and avoid complications (Figure 3).

Concerning future developments on this topic, differences in intestinal microbiota have already been found between patients who develop AL and patients who suffered no complications in the postoperative period. This may become a future therapeutic topic. Another target worth exploring in future studies is the role of MAT in this whole process. MAT’s resection demonstrably reduces disease recurrence and the need for reoperation in long-term follow-up. However, its influence on anastomotic healing and the relationship between the degree of mesenteric involvement and early postoperative complication rates in CD are two current research gaps that have yet to be addressed in the literature.

## References

[1] Na SY Moon W. Perspectives on current and novel treatments for inflammatory bowel disease. Gut Liver. (2019) 13(6):604–16. 10.5009/gnl19019 31195433 PMC6860034

[2] Fucilini LMP Genaro LM Sousa DCE Coy CSR Leal RF Ayrizono MLS. Epidemiological profile and clinical characteristics of inflammatory bowel diseases in a brazilian referral center. Arq Gastroenterol. (2021) 58(4):483–90. 10.1590/s0004-2803.202100000-87 34909854

[3] Slavu I Alecu L Tulin A Mihaila D Braga V Voiosu T Reintervention rate following emergency surgery for crohn disease. Chirurgia (Bucur). (2018) 113(2):227–33. 10.21614/chirurgia.113.2.227 29733016

[4] Fichera A Schlottmann F Krane M Bernier G Lange E. Role of surgery in the management of Crohn’s disease. Curr Probl Surg. (2018) 55(5):162–87. 10.1067/j.cpsurg.2018.05.001 29935551

[5] Group ESoCC. Predictors for anastomotic leak, postoperative complications, and mortality after right colectomy for cancer: results from an international snapshot audit. Dis Colon Rectum. (2020) 63(5):606–18. 10.1097/DCR.0000000000001590 32032201

[6] Sparreboom CL van Groningen JT Lingsma HF Wouters MWJM Menon AG Kleinrensink GJ Different risk factors for early and late colorectal anastomotic leakage in a nationwide audit. Dis Colon Rectum. (2018) 61(11):1258–66. 10.1097/DCR.0000000000001202 30239395

[7] Baelum JK Qvist N Ellebaek MB. Ileorectal anastomosis in patients with Crohn’s disease. Postoperative complications and functional outcome-a systematic review. Colorectal Dis. (2021) 23(10):2501–14. 10.1111/codi.15839 34309170

[8] Johnston WF Stafford C Francone TD Read TE Marcello PW Roberts PL What is the risk of anastomotic leak after repeat intestinal resection in patients with Crohn’s disease? Dis Colon Rectum. (2017) 60(12):1299–306. 10.1097/DCR.0000000000000946 29112566

[9] Resegotti A Astegiano M Farina EC Ciccone G Avagnina G Giustetto A Side-to-side stapled anastomosis strongly reduces anastomotic leak rates in Crohn’s disease surgery. Dis Colon Rectum. (2005) 48(3):464–8. 10.1007/s10350-004-0786-6 15719193

[10] Eder P Adler M Dobrowolska A Kamhieh-Milz J Witowski J. The role of adipose tissue in the pathogenesis and therapeutic outcomes of inflammatory bowel disease. Cells. (2019) 8(6):628–46. 10.3390/cells8060628 31234447 PMC6627060

[11] Li Y Mohan H Lan N Wu X Zhou W Gong J Mesenteric excision surgery or conservative limited resection in Crohn’s disease: study protocol for an international, multicenter, randomized controlled trial. Trials. (2020) 21(1):210. 10.1186/s13063-020-4105-x 32085793 PMC7035646

[12] Feng JS Li JY Yang Z Chen XY Mo JJ Li SH. Stapled side-to-side anastomosis might be benefit in intestinal resection for Crohn’s disease: a systematic review and network meta-analysis. Medicine (Baltimore). (2018) 97(15):e0315. 10.1097/MD.0000000000010315 29642162 PMC5908623

[13] Bemelman WA Warusavitarne J Sampietro GM Serclova Z Zmora O Luglio G ECCO-ESCP Consensus on Surgery for Crohn’s Disease. J Crohns Colitis. (2018) 12(1):1–16. 10.1093/ecco-jcc/jjx061 28498901

[14] Diseases BSGoIB. Consensus guidelines for the management of inflammatory bowel disease. Arq Gastroenterol. (2010) 47(3):313–25. 10.1590/S0004-28032010000300019 21140096

[15] Hota S Parascandola S Smith S Tampo MM Amdur R Obias V. Robotic and laparoscopic surgical techniques in patients with Crohn’s disease. Surg Endosc. (2021) 35(8):4602–8. 10.1007/s00464-020-07885-x 32789588

[16] Johnson CS Kassir A Marx DS Soliman MK. Performance of da Vinci Stapler during robotic-assisted right colectomy with intracorporeal anastomosis. J Robot Surg. (2019) 13(1):115–9. 10.1007/s11701-018-0828-z 29846869

[17] Crippa J Carvello M Kotze PG Spinelli A. Robotic surgery in inflammatory bowel disease. Curr Drug Targets. (2021) 22(1):112–6. 10.2174/1389450121999200820125918 33109059

[18] An V Cohen L Lawrence M Thomas M Andrews J Moore J. Early surgery in Crohn’s disease a benefit in selected cases. World J Gastrointest Surg. (2016) 8(7):492–500. 10.4240/wjgs.v8.i7.492 27462391 PMC4942749

[19] Zhu M Feng Q Xu X Qiao Y Cui Z Yan Y Efficacy of early intervention on the bowel damage and intestinal surgery of Crohn’s disease, based on the Lémann index. BMC Gastroenterol. (2020) 20(1):421. 10.1186/s12876-020-01575-7 33308166 PMC7733289

[20] Stevens TW Haasnoot ML D’Haens GR Buskens CJ de Groof EJ Eshuis EJ Laparoscopic ileocaecal resection versus infliximab for terminal ileitis in Crohn’s disease: Retrospective long-term follow-up of the LIR!C trial. Lancet Gastroenterol Hepatol. (2020) 5(10):900–7. 10.1016/S2468-1253(20)30117-5 32619413

[21] Singh S Al-Darmaki A Frolkis AD Seow CH Leung Y Novak KL Postoperative mortality among patients with inflammatory bowel diseases: a systematic review and eta-analysis of population-based studies. Gastroenterology. (2015) 149(4):928–37. 10.1053/j.gastro.2015.06.001 26055136

[22] Maruyama BY Ma C Panaccione R Kotze PG. Early laparoscopic ileal resection for localized ileocecal Crohn’s disease: hard sell or a revolutionary new norm? Inflamm Intest Dis. (2022) 7(1):13–20. 10.1159/000515959 35224013 PMC8820134

[23] Lightner AL Shen B. Perioperative use of immunosuppressive medications in patients with Crohn’s disease in the new “biological era”. Gastroenterol Rep (Oxf). (2017) 5(3):165–77. 10.1093/gastro/gow046 28852521 PMC5554387

[24] Fleshner P. Expert commentary on perioperative management of biologic and immunosuppressive medications in Crohn’s disease. Dis Colon Rectum. (2018) 61(4):431–2. 10.1097/DCR.0000000000001073 29521823

[25] Benchimol EI Seow CH Steinhart AH Griffiths AM. Traditional corticosteroids for induction of remission in Crohn’s disease. Cochrane Database Syst Rev. (2008) (2):CD006792. 10.1002/14651858.CD006792.pub2 18425970 PMC6718222

[26] Rezaie A Kuenzig ME Benchimol EI Griffiths AM Otley AR Steinhart AH Budesonide for induction of remission in Crohn’s disease. Cochrane Database Syst Rev. (2015) (6):CD000296. 10.1002/14651858.CD000296.pub4 26039678 PMC10613338

[27] Ford AC Bernstein CN Khan KJ Abreu MT Marshall JK Talley NJ Glucocorticosteroid therapy in inflammatory bowel disease: systematic review and meta-analysis. Am J Gastroenterol. (2011) 106(4):590–9, quiz 600. 10.1038/ajg.2011.70 21407179

[28] Steinhart AH Ewe K Griffiths AM Modigliani R Thomsen OO. Corticosteroids for maintenance of remission in Crohn’s disease. Cochrane Database Syst Rev. (2003) (4):CD000301. 10.1002/14651858.CD000301 14583917

[29] Subramanian V Saxena S Kang JY Pollok RC. Preoperative steroid use and risk of postoperative complications in patients with inflammatory bowel disease undergoing abdominal surgery. Am J Gastroenterol. (2008) 103(9):2373–81. 10.1111/j.1572-0241.2008.01942.x 18616660

[30] Anstead GM. Steroids, retinoids, and wound healing. Adv Wound Care. (1998) 11(6):277–85. 10326344

[31] Lightner AL. Perioperative management of biologic and immunosuppressive medications in patients with Crohn’s disease. Dis Colon Rectum. (2018) 61(4):428–31. 10.1097/DCR.0000000000001072 29521822

[32] Preiß JC Bokemeyer B Buhr HJ Dignaß A Häuser W Hartmann F [Updated German clinical practice guideline on “Diagnosis and treatment of Crohn’s disease” 2014]. Z Gastroenterol. (2014) 52(12):1431–84. 10.1055/s-0034-1385199 25474283

[33] Colombel JF Loftus EV Tremaine WJ Pemberton JH Wolff BG Young-Fadok T Early postoperative complications are not increased in patients with Crohn’s disease treated perioperatively with infliximab or immunosuppressive therapy. Am J Gastroenterol. (2004) 99(5):878–83. 10.1111/j.1572-0241.2004.04148.x 15128354

[34] Aberra FN Lewis JD Hass D Rombeau JL Osborne B Lichtenstein GR. Corticosteroids and immunomodulators: postoperative infectious complication risk in inflammatory bowel disease patients. Gastroenterology. (2003) 125(2):320–7. 10.1016/S0016-5085(03)00883-7 12891531

[35] Afzali A Park CJ Zhu K Hu JK Sharma P Sinanan MN Preoperative use of ethotrexate and the risk of early postoperative complications in patients with inflammatory bowel disease. Inflamm Bowel Dis. (2016) 22(8):1887–95. 10.1097/MIB.0000000000000780 27057681

[36] Torres J Mehandru S Colombel JF Peyrin-Biroulet L. Crohn’s disease. Lancet. (2017) 389(10080):1741–55. 10.1016/S0140-6736(16)31711-1 27914655

[37] Misselwitz B Juillerat P Sulz MC Siegmund B Brand S, Swiss IBDnet aowgotSSoG. Emerging treatment options in inflammatory bowel disease: Janus kinases, stem cells, and more. Digestion. (2020) 101(Suppl 1):69–82. 10.1159/000507782 32570252

[38] Scott FI. Infliximab versus biosimilars for IBD: is it better to fight than switch? Dig Dis Sci. (2020) 65(8):2158–60. 10.1007/s10620-020-06283-6 32338328

[39] Kienle P. Impact of modern drug therapy on surgery: Crohn’s disease. Visc Med. (2018) 34(6):422–5. 10.1159/000495127 30675486 PMC6341356

[40] Sciuto A Merola G De Palma GD Sodo M Pirozzi F Bracale UM Predictive factors for anastomotic leakage after laparoscopic colorectal surgery. World J Gastroenterol. (2018) 24(21):2247–60. 10.3748/wjg.v24.i21.2247 29881234 PMC5989239

[41] Zhu QL Feng B Lu AG Wang ML Hu WG Li JW Laparoscopic low anterior resection for rectal carcinoma: Complications and management in 132 consecutive patients. World J Gastroenterol. (2010) 16(36):4605–10. 10.3748/wjg.v16.i36.4605 20857534 PMC2945495

[42] Shah RS Bachour S Jia X Holubar SD Hull TL Achkar JP Hypoalbuminaemia, not biologic exposure, is associated with postoperative complications in Crohn’s disease patients undergoing ileocolic resection. J Crohns Colitis. (2021) 15(7):1142–51. 10.1093/ecco-jcc/jjaa268 33388775 PMC8427722

[43] Cambi MPC Yamamoto T Kotze PG. Importance of nutrition and hypoalbuminaemia in postoperative morbidity in Crohn’s disease: thinking outside of the box on biologics as single risk factors. J Crohns Colitis. (2021) 15(8):1401–2. 10.1093/ecco-jcc/jjab019 33508077

[44] Shimura T Toiyama Y Hiro J Imaoka H Fujikawa H Kobayashi M Monitoring perioperative serum albumin can identify anastomotic leakage in colorectal cancer patients with curative intent. Asian J Surg. (2018) 41(1):30–8. 10.1016/j.asjsur.2016.07.009 27451010

[45] Zhang Z Pereira SL Luo M Matheson EM. Evaluation of blood biomarkers associated with risk of malnutrition in older adults: a systematic review and meta-analysis. Nutrients. (2017) 9(8):829–48. 10.3390/nu9080829 PMC5579622 28771192

[46] Feng Y Li Y Mei S Zhang L Gong J Gu L Exclusive enteral nutrition ameliorates mesenteric adipose tissue alterations in patients with active Crohn’s disease. Clin Nutr. (2014) 33(5):850–8. 10.1016/j.clnu.2013.10.009 24200200

[47] Levine A Wine E Assa A Sigall Boneh R Shaoul R Kori M Crohn's disease exclusion diet plus partial enteral nutrition induces sustained remission in a randomized controlled trial. Gastroenterology. (2019) 157(2):440–50.e8. 10.1053/j.gastro.2019.04.021 31170412

[48] Guo Z Guo D Gong J Zhu W Zuo L Sun J Preoperative Nutritional Therapy Reduces the Risk of Anastomotic Leakage in Patients with Crohn’s Disease Requiring Resections. Gastroenterol Res Pract. (2016) 2016:5017856. 10.1155/2016/5017856 26858749 PMC4706910

[49] Yamamoto T Nakahigashi M Saniabadi AR Iwata T Maruyama Y Umegae S Impacts of long-term enteral nutrition on clinical and endoscopic disease activities and mucosal cytokines during remission in patients with Crohn’s disease: a prospective study. Inflamm Bowel Dis. (2007) 13(12):1493–501. 10.1002/ibd.20238 17879280

[50] Bischoff SC Escher J Hébuterne X Kłęk S Krznaric Z Schneider S ESPEN practical guideline: clinical Nutrition in inflammatory bowel disease. Clin Nutr. (2020) 39(3):632–53. 10.1016/j.clnu.2019.11.002 32029281

[51] Adamina M Bonovas S Raine T Spinelli A Warusavitarne J Armuzzi A ECCO guidelines on therapeutics in Crohn’s disease: surgical treatment. J Crohns Colitis. (2020) 14(2):155–68. 10.1093/ecco-jcc/jjz187 31742338

[52] Brennan GT Ha I Hogan C Nguyen E Jamal MM Bechtold ML Does preoperative enteral or parenteral nutrition reduce postoperative complications in Crohn’s disease patients: a meta-analysis. Eur J Gastroenterol Hepatol. (2018) 30(9):997–1002. 10.1097/MEG.0000000000001162 29738326

[53] Zulian A Cancello R Ruocco C Gentilini D Di Blasio AM Danelli P Differences in visceral fat and fat bacterial colonization between ulcerative colitis and Crohn's disease. An in vivo and in vitro study. PLoS One. (2013) 8(10):e78495. 10.1371/journal.pone.0078495 24205244 PMC3813471

[54] Amar J Chabo C Waget A Klopp P Vachoux C Bermúdez-Humarán LG Intestinal mucosal adherence and translocation of commensal bacteria at the early onset of type 2 diabetes: molecular mechanisms and probiotic treatment. EMBO Mol Med. (2011) 3(9):559–72. 10.1002/emmm.201100159 21735552 PMC3265717

[55] Thursby E Juge N. Introduction to the human gut microbiota. Biochem J. (2017) 474(11):1823–36. 10.1042/BCJ20160510 28512250 PMC5433529

[56] Elson CO Alexander KL. Host-microbiota interactions in the intestine. Dig Dis. (2015) 33(2):131–6. 10.1159/000369534 25925913

[57] Manichanh C Rigottier-Gois L Bonnaud E Gloux K Pelletier E Frangeul L Reduced diversity of faecal microbiota in Crohn’s disease revealed by a metagenomic approach. Gut. (2006) 55(2):205–11. 10.1136/gut.2005.073817 16188921 PMC1856500

[58] Seksik P Rigottier-Gois L Gramet G Sutren M Pochart P Marteau P Alterations of the dominant faecal bacterial groups in patients with Crohn’s disease of the colon. Gut. (2003) 52(2):237–42. 10.1136/gut.52.2.237 12524406 PMC1774977

[59] Yu YN Fang JY. Gut Microbiota and Colorectal Cancer. Gastrointest Tumors. (2015) 2(1):26–32. 10.1159/000380892 26674881 PMC4668798

[60] Lucke K Miehlke S Jacobs E Schuppler M. Prevalence of Bacteroides and Prevotella spp. in ulcerative colitis. J Med Microbiol. (2006) 55(Pt 5):617–24. 10.1099/jmm.0.46198-0 16585651

[61] Bosmans JW Jongen AC Boonen BT van Rijn S Scognamiglio F Stucchi L Comparison of three different application routes of butyrate to improve colonic anastomotic strength in rats. Int J Colorectal Dis. (2017) 32(3):305–13. 10.1007/s00384-016-2718-z 27942836 PMC5316396

[62] Yamada F Endo N Miyatake S Ebisu G Hino K. Enteral feeding with low-methoxyl pectin accelerates colonic anastomosis healing in rats. Nutrition. (2018) 45:94–8. 10.1016/j.nut.2017.07.013 29129243

[63] Limketkai BN Iheozor-Ejiofor Z Gjuladin-Hellon T Parian A Matarese LE Bracewell K Dietary interventions for induction and maintenance of remission in inflammatory bowel disease. Cochrane Database Syst Rev. (2019) 2:CD012839. 10.1002/14651858.CD012839.pub2 30736095 PMC6368443

[64] Gerasimidis K Nichols B McGowan M Svolos V Papadopoulou R Kokkorou M The effects of commonly consumed dietary fibres on the gut microbiome and its fibre fermentative capacity in adults with inflammatory bowel disease in remission. Nutrients. (2022) 14(5):1053–67. 10.3390/nu14051053 35268028 PMC8912623

[65] van Praagh JB de Goffau MC Bakker IS Harmsen HJ Olinga P Havenga K. Intestinal microbiota and anastomotic leakage of stapled colorectal anastomoses: a pilot study. Surg Endosc. (2016) 30(6):2259–65. 10.1007/s00464-015-4508-z 26385781 PMC4887536

[66] Glassner KL Abraham BP Quigley EMM. The microbiome and inflammatory bowel disease. J Allergy Clin Immunol. (2020) 145(1):16–27. 10.1016/j.jaci.2019.11.003 31910984

[67] Coffey JC O’Leary DP Kiernan MG Faul P. The mesentery in Crohn’s disease: friend or foe? Curr Opin Gastroenterol. (2016) 32(4):267–73. 10.1097/MOG.0000000000000280 27115218

[68] Peltrini R Bucci L. “Mesentery-based surgery” to prevent surgical recurrence in Crohn’s disease: from basics to surgical practice. Int J Colorectal Dis. (2019) 34(2):353–4. 10.1007/s00384-018-3197-1 30417271

[69] Coffey CJ Kiernan MG Sahebally SM Jarrar A Burke JP Kiely PA Inclusion of the mesentery in ileocolic resection for crohn’s disease is associated with reduced surgical recurrence. J Crohns Colitis. (2018) 12(10):1139–50. 10.1093/ecco-jcc/jjx187 29309546 PMC6225977

[70] Zhu Y Qian W Huang L Xu Y Guo Z Cao L Role of extended mesenteric excision in postoperative recurrence of Crohn’s colitis: a single-center study. Clin Transl Gastroenterol. (2021) 12(10):e00407. 10.14309/ctg.0000000000000407 34597277 PMC8483874

[71] Alshantti A Hind D Hancock L Brown SR. The role of Kono-S anastomosis and mesenteric resection in reducing recurrence after surgery for Crohn’s disease: a systematic review. Colorectal Dis. (2021) 23(1):7–17. 10.1111/codi.15136 32418300

[72] Kono T Fichera A. Surgical treatment for Crohn’s disease: a role of Kono-S Anastomosis in the West. Clin Colon Rectal Surg. (2020) 33(6):335–43. 10.1055/s-0040-1714236 33162837 PMC7605911

[73] Ng CH Chin YH Lin SY Koh JWH Lieske B Koh FH Kono-S anastomosis for Crohn’s disease: a systemic review, meta-analysis, and meta-regression. Surg Today. (2021) 51(4):493–501. 10.1007/s00595-020-02130-3 32894346

[74] Luglio G Rispo A Imperatore N Giglio MC Amendola A Tropeano FP Surgical prevention of anastomotic recurrence by excluding mesentery in Crohn’s disease: the SuPREMe-CD study - a randomized clinical trial. Ann Surg. (2020) 272(2):210–7. 10.1097/SLA.0000000000003821 32675483

